# Save our surgeons (SOS) – an explorative comparison of surgeons’ muscular and cardiovascular demands, posture, perceived workload and discomfort during robotic vs. laparoscopic surgery

**DOI:** 10.1007/s00404-022-06841-5

**Published:** 2022-11-19

**Authors:** Bernhard Krämer, Felix Neis, Christl Reisenauer, Christina Walter, Sara Brucker, Diethelm Wallwiener, Robert Seibt, Julia Gabriel, Monika A. Rieger, Benjamin Steinhilber

**Affiliations:** 1 Department of Women’s Health, University Hospital Tübingen, Calwerstr 7/6, 72076 Tübingen, Germany; 2 Institute of Occupational and Social Medicine and Health Services Research, University Hospital Tübingen, University of Tübingen, Wilhelmstraße 27, 72074 Tübingen, Germany

**Keywords:** Robotic-assisted surgery, Ergonomics, Surface electromyography, Static muscle demands, Musculoskeletal discomfort, Work-related musculoskeletal disorders

## Abstract

**Purpose:**

Conventional laparoscopic surgery (CLS) imposes an increased risk of work-related musculoskeletal disorders. Technical innovations, such as robotic-assisted laparoscopic surgery (RALS), may provide ergonomic benefits. We compare the surgeon`s work-related demands of CLS vs RALS for benign hysterectomies.

**Methods:**

Five specialists (3 females, 2 males) each performed four RALS and four CLS as part of their daily clinical routine. During the surgical procedures, muscular demands were assessed by bipolar surface electromyograms of the descendent trapezius, extensor digitorum and flexor carpi radialis muscles as well as cardio-vascular demands by electrocardiography, and neck, arm and torso posture by gravimetrical position sensors. Additionally, the subjects rated their level of perceived workload (NASA TLX questionnaire with 6 dimension) and musculoskeletal discomfort (11-point Likert-scale, 0–10).

**Results:**

Muscular demands of the trapezius and flexor carpi radialis muscles were lower with RALS but extensor digitorum demands increased. Cardiovascular demands were about 9 heart beats per minute (bpm) lower for RALS compared to CLS with a rather low median level for both surgical techniques (RALS = 84 bpm; CLS 90 bpm). The posture changed in RALS with an increase in neck and torso flexion, and a reduction in abduction and anteversion position of the right arm. The perceived workload was lower in the physical demands dimension but higher in the mental demands dimension during RALS. Subjective musculoskeletal discomfort was rare during both surgical techniques.

**Conclusions:**

This explorative study identified several potential ergonomic benefits related to RALS which now can be verified by studies using hypothesis testing designs. However, potential effects on muscular demands in the lower arm extensor muscles also have to be addressed in such studies.

**Supplementary Information:**

The online version contains supplementary material available at 10.1007/s00404-022-06841-5.

## What does this study add to the clinical work

**Table:** 

This study focuses on the comparison of ergonomic parameters between laparoscopic hysterectomy and robotic-assisted hysterectomy. It contributes valuable information for the assessment of potential benefits with respect to surgeon`s discomfort and occupational health.	

## Introduction

Work-related musculoskeletal disorders (WRMSDs) among surgeons are problems that often lack awareness or are neglected by individuals in often highly competitive surgical settings. Degenerative lumbar spine disease (19%), rotator cuff pathology (18%), degenerative cervical spine disease (17%), followed by carpal tunnel syndrome (9%) are mostly diagnosed. Even higher prevalence levels are found for musculoskeletal pain, with estimated 12-month prevalence levels of 60% for neck pain, 52% for shoulder pain, 49% for back pain and 35% for the pain of the upper extremities [1]. Surgeons performing minimally invasive surgery (MIS) are at particular risk [2–4]; the prevalence of WRMSDs in this employee group may be up to 74% as indicated by a systematic review [4]. Typically, the neck, the back, shoulders, wrist, hands and thumbs are affected [3, 5, 6].

Robotic surgery has gained popularity and significance in MIS over the last few years and clinicians hope to further expand on the recognized advantages of the laparoscopic surgical technique for patients [7]. In this context, many scientific papers have reported on patient outcomes and surgical data when applying robotic surgery which has been summarized in recent systematic reviews [8, 9]. In addition, it is presumed that robotic surgery provides ergonomic benefits for surgeons by reducing physical demands [10]. However, the influence of robotic-assisted surgery on reducing physical exposures in MIS is inconclusive. Some authors report less physical discomfort in surgeons performing robotic-assisted surgery compared to laparoscopic surgery [11, 12], while others found higher muscular effort and no differences in muscular fatigue [13]. Catanzarite and colleagues 2018 pointed out the importance of better characterizing the ergonomic risks and benefits associated with this surgical technique since the number of robotic-assisted procedures is increasing [14].

The present study, therefore, exploratively investigated ergonomic aspects in terms of surgeons’ muscular and cardiovascular demands, neck, arm and torso posture as well as perceived workload and musculoskeletal discomfort during standard hysterectomy procedures -performed by robotic-assisted laparoscopic surgery (RALS) versus conventional laparoscopic surgery (CLS).

## Material and methods

### Subjects

Five surgeons, two males (BK, FN) and three females (SB, CW, CR) with experience in minimally invasive gynaecology participated in the present study. All of them had previously performed ≥ 10 RALS to achieve a stable competence level in the learning curve and to show a comparable routine with regard to CLS. Further inclusion criteria were inconspicuous orienting functional physical examination of the upper extremities, ability to work in a full shift, voluntary participation and a signed written informed consent. The study was approved by the Ethics Committee of the Medical Faculty and the University Hospital of Tübingen, Germany (262/2018BO1) and was registered under ClinicalTrial.gov (NCT04352452).

#### Procedures

Each surgeon performed four RALS and four CLS according to their daily clinical routine from May 2020 to May 2021. The order of surgeries was not randomized but took place as per the schedule of the department. The exact sequence of operations and measurement days for each surgeon were documented and are provided as supplemental material (SUPPLEMENT A). The investigated procedure was a laparoscopic hysterectomy for benign indications. This is a routine procedure that is frequently performed and which can be done as CLS or RALS. Four surgeons performed eight hysterectomies each (4 RALS and 4 CLS per individual) except for one who performed 4 subtotal (supracervical) hysterectomies with subsequent cervico-colposacropexy with each technique (4 RALS and 4 CLS).

The RALS were conducted with the DaVinci-SI-system (Da Vinci SI; Intuitive Surgical Ltd., Sunnyvale, California, USA) for which bipolar forceps (left port), monopolar scissors and a needle holder (each docked through one right port) were used. After the insertion of an intrauterine manipulator (Manipulator n. Hohl, Karl Storz, Tuttlingen, Germany), an assistant sat between the patient`s legs and guided the tool according to the console surgeon`s instructions to present the preparation planes. No further assistant participated except during the hysterectomies with subsequent cervico-colposacropexy, which engaged one more assistant exclusively for the two steps of uterus morcellation and the fixation of the mesh on the promontory, for which an additional trocart was placed in the lower abdomen. The ergonomic burden of the assistants was not analyzed.

The vaginal cuff was closed robotically with a running suture (V-Loc 180, 0, 9″ or 12″, Covidien, Mansfield, USA).

For the CLS, the hospital`s standard setting with surgeon (positioned at the left side of the patient), first assistant (right side) and second assistant (manipulator, see above) with the following trocart placements were used: a grasper (left trocart), another grasper for the assistant (right trocart) as well as a midline trocart between the symphysis and the umbilicus for the alternate insertion of bipolar coagulation forceps and scissors.

In this group, the vaginal cuff was closed vaginally with interrupted sutures (Vicryl CT 1 plus, Ethicon, Johnson&Johnson International, Diegem, Belgium).

### Measurements and data analysis

**Fig. 1:**
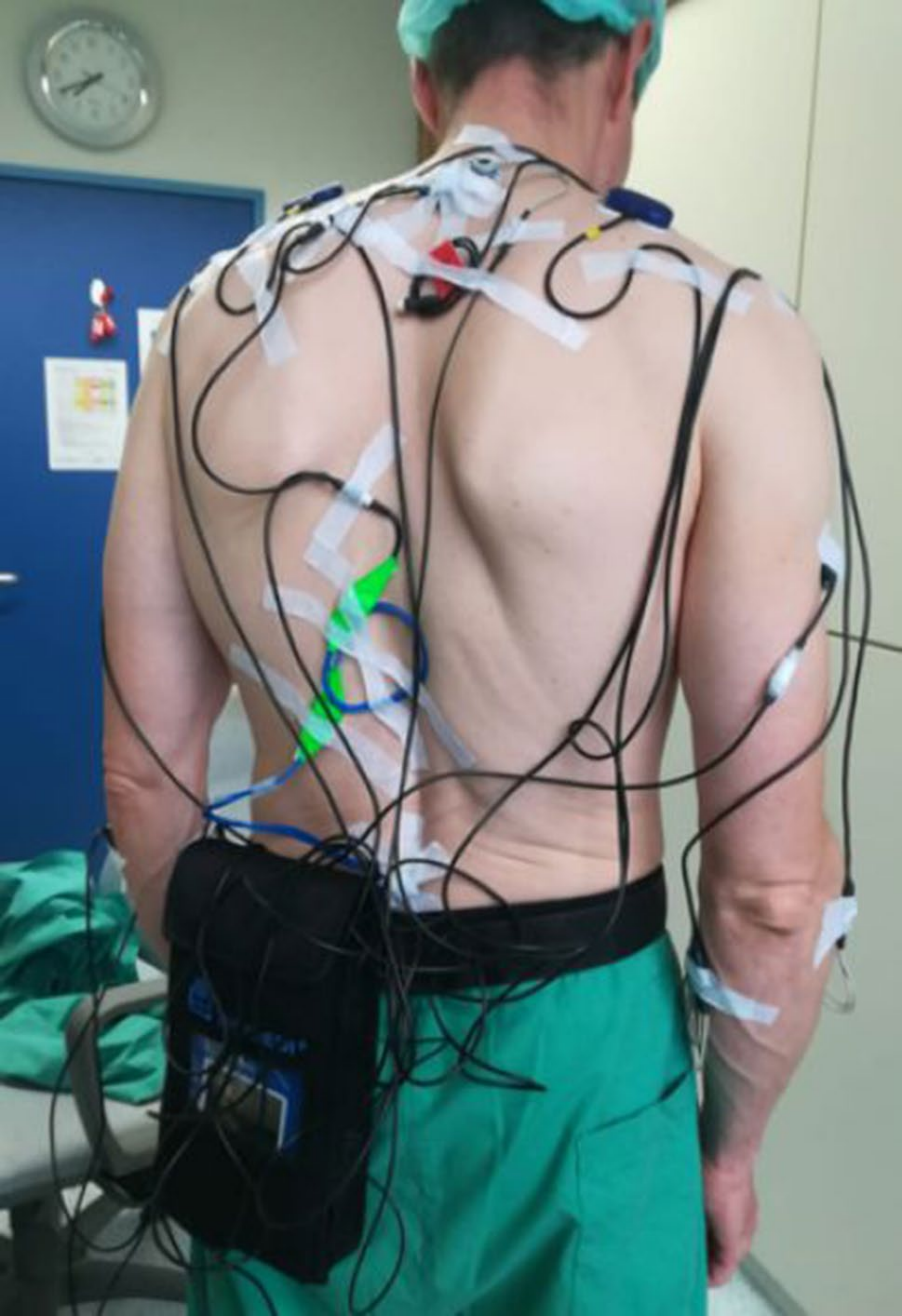
Surgeon equipped with the measurement device for assessing the laparoscopic procedures

During the surgical steps for benign hysterectomy (i.e. the electrocoagulation and cutting of the tissue, the preparation of the planes, colpotomy and vaginal cuff closure) surface-electromyography (sEMG), electrocardiography (ECG) and posture data of the surgeons were collected continuously using a PS12-II device (THUMEDI® GmbH & Co. KG, Thum, Germany) which was worn on the body throughout the procedures (Fig. 1). The incisions for trocart placements and the suture of the skin were not measured. The perceived level of musculoskeletal discomfort was rated every 20 min by the surgeons and at the very end of the surgical procedure with respect to affected body areas. Immediately after the completion of their surgical procedure the surgeons were asked to rate their perceived workload and to assess the difficulty of the surgery and their perceived working precision regarding the applied surgical technique.

*Muscular demands*: Bipolar surface electromyography (sEMG) was used to measure muscle activities of both descending trapezius muscles (TD), lower arm extensor digitorum muscles (ED) and flexors carpi radialis muscles (FCR). The skin was cleaned with an abrasive paste (Nuprep skin preparation gel, Weaver and Company, USA) and shaved if there was excessive body hair. Electrodes were self-sticking silver/silver chloride (Ag/AgCl) electrodes with an active diameter of 15 mm and an inter-electrode distance of 25 mm. The EMG signal was differentially amplified, transmitted, filtered (high-pass filter, 2nd order, − 3 dB, 4 Hz; low-pass filter, 11th order, − 3 dB, 1300 Hz), sampled (4096 Hz), analyzed and stored (PS12-II, THUMEDI® GmbH & Co. KG, Thum, Germany; physical resolution 24bit; common-mode rejection ration > 98 dB, effective sum of noise < 0.5 µV RMS; linearity ± 0.1 dB at 30–1200 Hz). The data was real-time transformed in the frequency domain (1024-point fast fourier transformation, 250-ms Bartlett window, 50% overlap) and digitally filtered (high-pass filter, 11th order, − 3 dB, 16 Hz). Interfering powerline noise was removed by an average filter (11th order, − 3 dB, 50 Hz and its first seven harmonics, 4-Hz bandwidth was replaced by spectral neighbours). The root-mean-square of electrical activity (RMS [μV]) was real-time calculated from the power spectrum (250-ms moving window, 50% overlap) and stored synchronously with the raw data. The RMS of each muscle was normalized to the 90th percentile of the RMS of the most stable 3-s period of an isometric maximum voluntary contraction (MVC) and expressed as a percent of the muscle’s maximum voluntary electrical activation (%MVE). Details about these procedures can be found in the supplemental material (SUPPLEMENT B). The normalized RMS values were further used to calculate the three outcome measures representing muscular demands: the 50th and 10th percentiles electrical activity of the surgical procedures and the muscular rest time defined as the proportion of muscle activation below 0.5%MVE during a surgical procedure. All of these three outcomes are associated with the risk of WRMSDs [15, 16].

*Neck, arm and torso posture:* 2D gravimetric position sensors (sampling rate 8 Hz; resolution of 0.1° and 125 ms in time; maximum static error of 0.5° against the perpendicular; maximum repetition error of 0.2°) continuously recorded inclination angles with respect to the absolute perpendicular (gravitational axis) as flexion and lateral flexion. The sensors were attached to the skin at the spinous processes of thoracic vertebrae one (T1) and lumbar vertebrae 4 (L4), the lateral part of the upper arm and the chin using double-sided adhesive tape (25 mm × 5 m; 3 M transparent Medical Standard, Top Secret®, Gesellschaft für Haarästhetik mbH, Fürth, Germany). The sensors measured the inclination in degrees towards the perpendicular line with respect to the sagittal plane (*x*-value) and frontal plane (*y*-value). Each measurement signal was offset adjusted according to the reference posture recorded prior to the experimental conditions by subtraction. During the reference posture, the inclination angles of sensors were recorded while each subject was standing in their common upright standing posture with the arms hanging down vertically and looking ahead for about 20 s. The median of the most stable 10 s within this period was defined as the reference. Neck, arm and torso postures were then calculated as differences between two sensors and were given as means per surgical procedure. The details of the calculation are provided in the supplemental material (SUPPLEMENT C).

*Cardiovascular demands*: As an indicator of cardiovascular demands the electrical activity of the heart was recorded using electrocardiography (ECG) by two pre-gelled Ag/AgCl electrodes placed ~ 5 cm cranial and ~ 3 cm left-lateral from the distal end of the sternum and over the anterior to mid-axillary line at the fifth left rib. ECG signals were continuously recorded (sample rate 1000 Hz) and processed in real-time to calculate heart rate (HR [bpm]) and was given as median per surgical procedure.

*Musculoskeletal discomfort:* The level of perceived musculoskeletal discomfort was rated by the surgeons using an 11-point Likert scale (0 = no discomfort, 10 maximum discomfort). The scale has been validated for assessing pain showing excellent reliability (intra-class coefficient of 0.95) and good to excellent validity in comparison with a visual analogue scale or verbal rating scale (Pearson correlation coefficient of 0.94 or 0.93) [17]. Although it has not been specifically validated for assessing musculoskeletal discomfort, it is commonly used in occupational settings [18]. In addition, subjects were able to indicate in which body region the discomfort was perceived (neck, shoulder, upper arm, elbow, lower arm, wrist, finger(s), upper back, lower back, or others). These ratings were used to calculate the relative frequency of perceived discomfort as the number of discomfort divided by the number of discomfort ratings per surgical procedure and were given in percent. In addition, the mean discomfort intensity was calculated with respect to the affected body area.

*Perceived workload:* Workload was assessed in six dimensions on scales with 21 gradations [0–20] using the NASA-TLX questionnaire. The dimensions are mental demand, physical demand, temporal demand (very low to very high), performance (perfect to failure), effort and frustration (very low to very high) and were analyzed separately. This questionnaire is an established and well-accepted tool in ergonomic research [19].

*Supplemental data: *The Nordic Questionnaire [20] was used to collect surgeons’ body weight, body height, age, gender, laterality, years of experience in surgery, their participation in regular physical activity and musculoskeletal complaints during the last 12 months. In addition, participants subjectively rated the difficulty of the surgery and their perceived working precision of the surgical technique by a visual analogue scale [0–100 mm] and the plain operation times of each surgical procedure without incision, suturing and preparing the technical devices were recorded.

### Statistical analysis

All outcome variables were visually inspected for extreme values and missing data. Means and standard deviations or boxplots including median and the upper and lower quartiles and interquartile ranges or frequencies were used to describe the results. Data were analyzed using JMP 16 (SAS Inc. Cary, NC, USA). For the outcomes, median electrical activity, 10th percentile electrical activity, muscular rest time, heart rate, neck, arm and torso posture, perceived workload, perceived task difficulty and precision as well as operation time, intra-individual differences between the two surgical techniques were calculated. In this regard, the absolute differences (*delta*) between the first RALS and CLS, second RALS and CLS, third RALS and CLS, and fourth RALS and CLS were determined leading to four absolute differences per subject and outcome variable. Differences (*delta*) between all RALS and CLS were calculated accordingly (*RALS*_*i*_ and *CLS*_*i*_, where *i* = *1 to n and n* = *20*).

1 $${\text{delta}}\left( {{\text{outcome}}} \right)_{i} = {\text{outcome}}_{{RALS_{i} }} - {\text{outcome}}_{{CLS_{i} }}$$

In the case of the median (*med*) and 10th percentile *(Perc.10)* normalized electrical activity relative differences (*delta*_*rel*_ (eA)) between RALS and CLS were calculated according to the following formula to account for the activity level in muscle demand:

2 $${\text{delta}}_{{\text{rel }}} \left( {eA_{{{\text{med}}}} } \right)_{i} = \frac{{eA_{{{\text{med}}}} \left( {RALS_{i} } \right) - eA_{{{\text{med}}}} \left( {CLS_{i} } \right)}}{{\left[ {\frac{{eA_{{{\text{med}}}} \left( {RALS_{i} } \right) + eA_{{{\text{med}}}} \left( {CLS_{i} } \right)}}{2}} \right]}}$$

3 $${\text{delta}}_{rel } \left( {eA_{Perc.10} } \right)_{i} = \frac{{eA_{Perc.10} \left( {RALS_{i} } \right) - eA_{Perc.10} \left( {CLS_{i} } \right)}}{{\left[ {\frac{{eA_{Perc.10} \left( {RALS_{i} } \right) + eA_{Perc.10} \left( {CLS_{i} } \right)}}{2}} \right]}}$$

Normal distribution of these differences was verified according to limits in standard errors of skewness and kurtosis suggested by [21]. A two-sided *t*-test or in case of non-normal distribution a nonparametric Wilcoxon–Mann–Withney Test was used to test whether the differences statistically deviate from zero (alpha level: 0.05). In addition, effect sizes were calculated for these measures according to Eqs. 1 or 2.

Cohen’s d was used for normally distributed differences or relative differences. Effect sizes from 0.2 to 0.5 are considered as a small effect, 0.5–0.8 as a medium effect, and > 0.8 corresponds to a large effect.

4 $${\text{Cohen' sd}} = \frac{{{\text{mean }}({\text{outcome}}_{{RALS_{i} }} - {\text{outcome}}_{{CLS_{i} }} )}}{{\sqrt {\frac{{\sum\nolimits_{{i = 1}}^{n} {\left\{ {({\text{outcome}}_{{RALS_{i} }} - {\text{outcome}}_{{CLS_{i} }} ) - {\text{mean }}({\text{outcome}}_{{RALS_{i} }} - {\text{outcome}}_{{CLS_{i} }} )} \right\}^{2} } }}{{n - 1}}} }}$$

where i = 1—n.

In the case of non-normally distributed differences or relative differences r-values according were calculated [22].

5 $$r = \left| {\frac{z}{{\sqrt {2 \times N} }}} \right|$$

The *z* values were derived from Wilcoxon tests comparing the two surgical techniques using the statistical software SPSS. *R* values from 0.1 to 0.3 indicate a small effect, values between 0.3 and 0.5 are considered as a medium effect and a large effect is associated with values over 0.5 [22].

The outcomes of musculoskeletal discomfort (relative discomfort frequency and discomfort intensity per body region) were analyzed using descriptive methods.

## Results

### Participants

None of the subjects dropped out of the study and eight surgeries (4 RALS, 4 CLS) were observed in each subject leading to an overall number of 40 surgical procedures that could be analyzed. The characteristics of the study population are given in Table 1. The subjects were all right-handed and none-smokers with a median of 19 years of experience in CLS.

**Table 1:** Characteristics of study population

Gender	2 Males
	3 Females
Age (years)	Median 45
	Interquartile range 13
Body weight (kg)	Median 70.0
	Interquartile range 30
Body height (cm)	Median 174
	Interquartile range 24
Body Mass Index (kg/m^2^)	Median 21.3
	Interquartile range 4.1
Regular physical activity (*n*)	Yes = 3
	No = 2
Smokers (*n*)	0
Experience in laparoscopic surgery (years)	Median 19
	Interquartile range 11.5
Laterality	All right handed

A one-year period of data acquisition was necessary to complete all measurements which was due to the COVID-19 pandemic that required the postponement of scheduled elective surgeries and thus expansion of the analysis period. As mentioned, the order of the surgical procedures was not randomized and the number of measurement days varied between subjects. The subject with the most experimental days was analyzed on eight separate days with only one surgery per day. The subject with the lowest number of experimental days was monitored on five days with three, two and three times one surgical procedure per day. Details can be found in the supplemental material (SUPPLEMENT A).

### Duration of the surgical procedures

No differences between the two surgical techniques were measured. CR`s procedures took longer, as a cervico-colpo-sacropexy was combined with the hysterectomies. The mean difference in duration between the two surgical techniques was 0.09 min ± 35.5 min. Note: the time for preparing the patients and the robotic device (draping, installing, docking, etc.) was not considered in this comparison. Details can be found in Table 2.

**Table 2:** Overview of all outcome variables with respect to the applied operation method

Parameter				RALS			CLS			Effect size Wilcoxon’s r (non normal distributed data)		Effect size Cohen’s d (normal distributed data)	
				Q1	Median	Q3	Q1	Median	Q3				
Muscle activity	Median electrical activity	Trapezius descendens muscle activity [%MVE]	Left	3.17	4.17	7.34	3.13	4.39	7.61			0.02	Min
			Right	3.23	4.43	6.98	7.45	10.73	13.01	0.80	Large		
		Extensor digitorum muscle activity [%MVE]	Left	5.62	10.51	21.73	6.55	7.76	11.27	0.52	Large		
			Right	4.12	9.00	15.03	7.12	10.91	13.35			0.01	Min
		Flexor carpi radialis muscle activity [%MVE]	Left	2.40	3.70	7.01	2.77	5.58	8.12			0.48	Small
			Right	1.31	3.18	6.37	2.35	5.59	7.18			0.88	Large
	10th percentile electrical activity	Trapezius descendens muscle activity [%MVE]	Left	0.66	1.06	1.43	1.20	1.73	3.39	0.63	Large		
			Right	0.38	0.52	0.94	3.22	5.26	7.10	0.88	Large		
		Extensor digitorum muscle activity [%MVE]	Left	3.11	5.38	10.37	2.45	3.28	4.84	0.62	Large		
			Right	2.24	3.47	6.68	2.18	3.68	4.79	0.35	Me-dium		
		Flexor carpi radialis muscle activity [%MVE]	Left	0.84	1.19	3.26	0.91	1.58	3.53			0.28	Small
			Right	0.45	0.95	1.99	0.65	1.62	2.27	0.61	Large		
Posture	Median angle	Neck flexion [°]	9.30	12.00	18.85	-6.90	-2.50	3.65			1.11	Large	
		Neck lateral flexion [°]	-3.89	1.83	6.90	-3.01	1.05	7.54			0.12	Min	
		Torso flexion [°]	8.20	14.00	21.80	3.04	5.45	13.05			0.71	Large	
		Torso lateral flexion [°]	-1.05	1.20	8.90	0.83	2.83	4.53			0.10	Min	
		Arm abduction [°]	left	2.75	7.65	14.81	3.98	7.35	8.99			0.08	Min
			Right	0.20	3.40	9.75	2.73	10.80	19.69	0.81	Large		
		Arm anteversion [°]	Left	-10.18	-6.20	7.08	-15.71	-11.58	2.95			0.23	Small
			Right	-11.00	-6.70	1.25	0.16	5.65	13.90			0.89	Large
OP duration	[min]			46.34	56.16	114.5	49.18	67.11	103.4		0.002	Min	
Heart rate	[1/min]			76.25	84.00	88.75	82.50	90.00	94.00		0.69	Large	
Difficulty	[0 – 100 mm]			32	57	75	22	31	74		0.36	Small	
Precision	[0 – 100 mm]			86	91	95	80	87	93		0.42	Small	
NASA-TLX	Mental demand [0 – 20]			10.00	14.00	15.50	6.50	10.00	14.00		0.43	Small	
	Physical demand [0 – 20]			4.50	9.50	10.00	8.00	10.00	12.75		0.41	Small	
	Temporal demand [0 – 20]			2.00	4.00	9.50	2.25	4.00	10.00	0.11	Small		
	Performance [0 – 20]			14.75	18.00	18.00	16.00	18.00	18.00	0.03	Min		
	Effort [0 – 20]			5.625	10.00	12.00	8.50	10.00	13.75			0.29	Small
	Frustration [0 – 20]			1.00	4.00	10.00	1.25	5.00	9.50	0.02	min		
Muscular Rest time													
(RMS < 0.5% MVE)	[% length of LP]	Trapezius descendens [%]	Left	2.39	3.76	8.26	0.38	1.31	4.14			0.56	Large
			Right	4.76	10.24	13.93	0.01	0.06	0.46			1.22	Large
		Extensor digitorum [%]	Left	0.00	0.01	0.08	0.00	0.01	0.65	0.16	Small		
			Right	0.00	0.40	1.65	0.00	0.06	1.20	0.05	Small		
		Flexor carpi radialis [%]	Left	0.03	0.20	5.10	0.00	0.03	4.14	0.46	Me-dium		
			Right	0.43	2.33	16.36	0.00	0.004	2.84			0.28	Small

Q1 1th quartile; Q3 3th quartile; *MVE* maximum voluntary electrical activation, *LP* laparoscopic procedure, *RALS* robotic assisted laparoscopic surgery, *SLS* standard laparoscopic surgery

### Perceived working precision and difficulty of the surgical procedures

The perceived *difficulty* of the surgical procedures was rated to be similar for RALS and CLS with a *non-significant* mean difference of 11 ± 30.2 mm (*p* = 0.13) on the visual analogue scale (0–100 mm). In both surgical techniques very low and very high levels of task difficulty were rated (RALS: Q1 32 mm, Median = 57 mm, Q3 75 mm; CLS: Q1 22 mm, Median = 31 mm, Q3 74 mm). Details are depicted in Table 2.

The difference in the perceived working precision was 3.4 ± 8.1 mm on the visual analogue scale (0–100 mm) and *did not show statistical significance* (*p*-value of 0.07). In both surgical techniques, surgeons rated high levels of working precision (RALS: Median = 91 mm; CLS: Median = 87 mm). Details are displayed in Table 2.

### Muscle activity – median and static muscular demands and muscular rest time

**Fig. 2:**
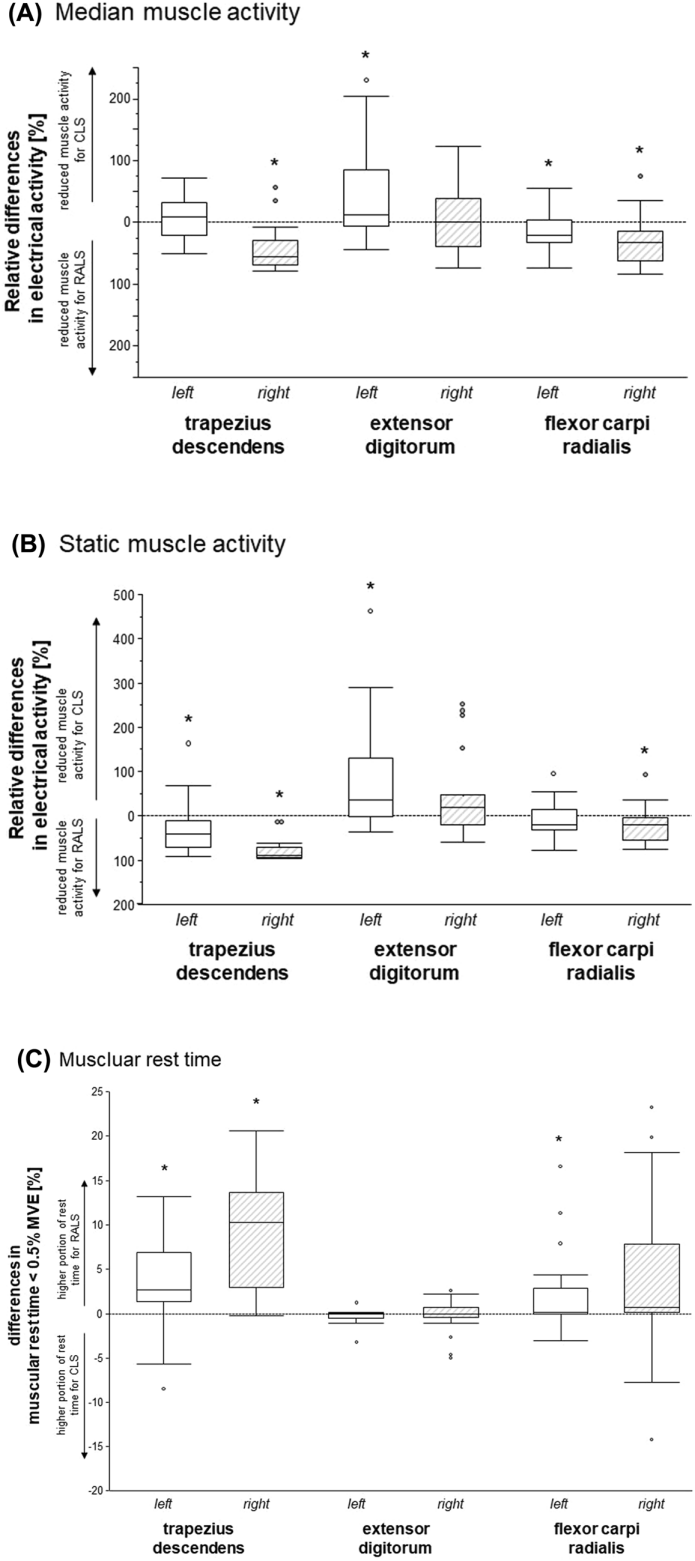
Differences muscular demands between standard and robotic-assisted laparoscopic surgery. *SLS* standard laparoscopic surgery, *RALS* robotic assisted laparoscopic surgery. **A** differences in median muscle activity. **B** differences in static muscle activity. **C** Differences in muscular rest time; Asterisks indicate statistically significant (alpha level 0.05) differences between standard and robotic assist laparoscopy

The comparison of muscle activity between the two surgical techniques indicated the potential for reducing muscular demands in the trapezius and the flexor carpi radials muscles when RALS is performed. However, the analysis also identified a risk for increasing muscular demands in the extensor digitorum muscle. In this respect, the three outcomes of muscular demands showed rather similar results (Fig. 2A–C). See Table 2.

Muscular demands of the trapezius muscles were characterized by *statistically significant* reductions in the static component of the muscle activity (Fig. 2B) and an increase in the proportion of muscular rest time (Fig. 2C) for the left and right trapezius muscles for RALS. The median electrical activity was only statistically significantly reduced in the right trapezius muscle (Fig. 2A).

With respect to the flexor carpi radialis muscle, applying RALS reduced the median electrical activity at both sides (Fig. 2A), while the static muscle activity was only reduced on the right side (Fig. 2B) and the proportion of muscular rest time was increased at the left lower arm flexor muscle only (Fig. 2C). Note: in three surgeries, the surface electrodes of the left flexor carpi radialis muscle were detached and the muscular demand outcomes could not be calculated.

The median and static activity of the left extensor digitorum muscle were *statistically significantly* increased (Fig. 2A and B) without a significant difference in muscular rest time (Fig. 2C) when RALS was performed.

### Cardiovascular demand

The heart rate *statistically significantly* decreased by 8.9 ± 12.2 beats per minute when surgeons performed RALS compared to CLS. The median heart rate level for RALS was 84 and for CLS 90 beats per minute. Details are displayed in Table 2.

### Neck, arm and torso posture

**Fig. 3:**
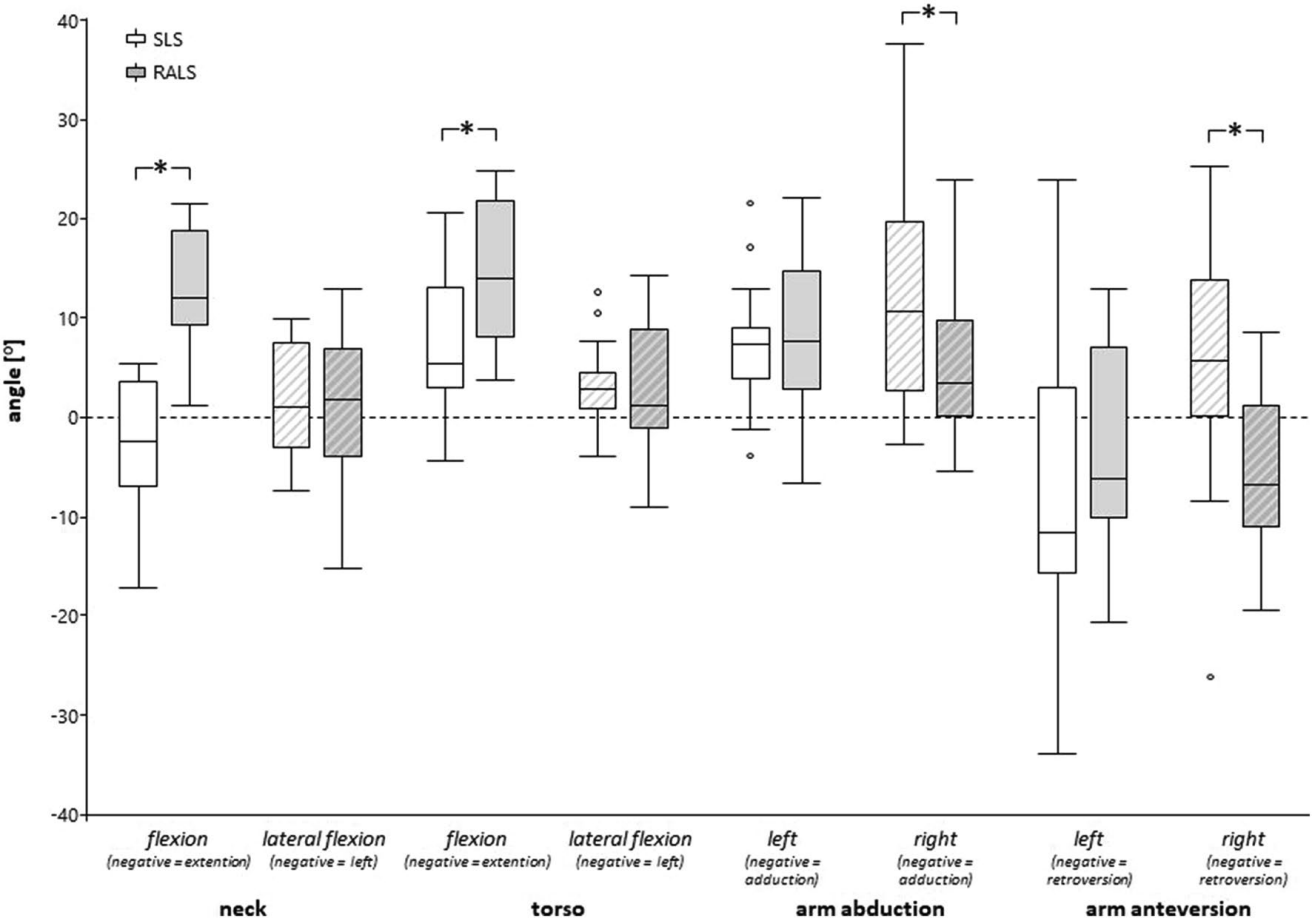
Differences in neck, torso and arm postures between standard and robotic-assisted laparoscopic surgery. *SLS *standard laparoscopic surgery, *RALS* robotic-assisted laparoscopic surgery; Asterisks indicate statistically significant (alpha level 0.05) differences between standard and robotic assist laparoscopy

The analysis of surgeons’ neck, arm, and torso postures indicated increased neck and torso flexion as well as reduced arm abduction and anteversion of the right arm when the RALS technique was applied. Lateral flexion of the neck and torso as well as arm posture of the left arm did not statistically differ between the two surgical techniques (Fig. 3). Details are depicted in Table 2.

### Perceived workload

**Fig. 4:**
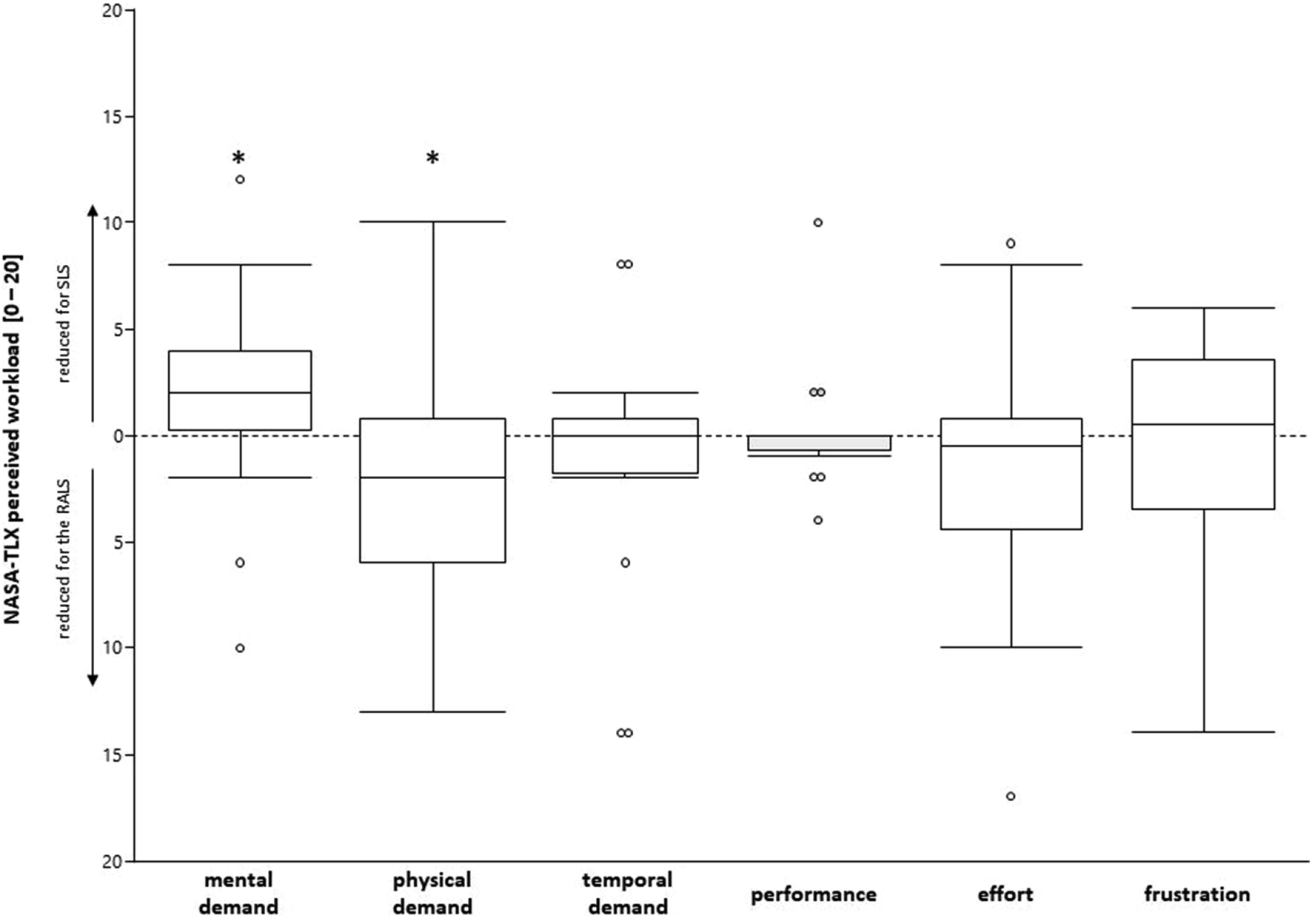
Differences in perceived workload between standard and robotic-assisted laparoscopic surgery. *SLS* standard laparoscopic surgery, *RALS* robotic-assisted laparoscopic surgery; Asterisks indicate statistically significant (alpha level 0.05) differences between standard and robotic assist laparoscopy

The six dimensions of perceived workload according to the NASA TLX questionnaire were analyzed separately and showed a decrease in perceived physical demands and an increase in mental demands in RALS in comparison to CLS (Fig. 4). No statistically significant difference occurred in the other four dimensions of perceived workload. Details are presented in Table 2.

## Discussion

Robotically assisted laparoscopic (RALS) procedures have increasingly entered the field of gynecological subspecialities in recent years. Technically, the indications that had been performed in a standard laparoscopic setting such as endometriosis, uro-gynaecology as well as radical or benign hysterectomies can also be mastered with robotics [23]. As a consequence, new (hybrid) robotic systems are entering the market. Data has shown that robotic surgery can be beneficial for selected patients [24]. In contrast, it is unclear whether RALS also has a significantly positive effect on ergonomics and subsequently on a surgeon`s risk for (work-related) musculoskeletal disorders [(WR)MSD]. The latter might be one of several reasons why a lack of active surgeons is expected in the near future [25, 26]. Accordingly, under the assumption that robotics may help to reduce WRMSD, valid data is necessary for hospitals` CEOs and controllers to justify the implementation of relatively costly robotic systems with the intention of maintaining or even attracting a stable number of surgical staff and to fulfil their potential fiduciary duties. Unfortunately, occupational health issues seem to be irrelevant to a high number of surgeons, as demonstrated by Tijam and colleagues who reported that about one-third of the 285 surveyed urologists had limited knowledge about ergonomics [3].

In addition to an increased workload of minimally invasive procedures, WRMSDs also arise from the position of the monitor, the use of foot pedals, the poorly adjusted operating table height, and the hand-held instruments [27], since they induce static and awkward body postures, sustained static muscular loading and non-neutral joint angles.

Against the background of these aspects, the rationale of our SOS-study was to exploratively compare the surgeons’ muscular and cardiovascular demands, neck, arm and torso posture as well as the perceived workload of conventional laparoscopic hysterectomy (CLS) versus RALS.

### Muscular demands

Our investigation indicated a potential benefit for the *trapezius and flexor carpi radialis muscle* by RALS but not for the extensor digitorum muscle. These results are in line with a review article from 2018. The authors also reported some advantages of RALS regarding neck and shoulder strain but also mentioned remaining strain in the wrist and fingers [14] which is associated with extensor muscle activity. Since minimally invasive surgeons specifically cite the neck as an area of musculoskeletal discomfort [28] which is associated with the trapezius muscle, a relief through RALS would be a significant improvement compared to the current situation in CLS. However, complaints in the hand area should of course not be worsened by RALS and need further assessments. In this respect, the developers of robotic devices are challenged to reduce possible risks through an ergonomic optimization of the hand-arm position or the handle design which has been shown to be possible for a number of laparoscopic instruments [29–31].

Previous studies have mainly observed the mean or median muscle activity between RALS and CLS when using sEMG [32]. Here, our study provides significant insight using three sEMG parameters, all of which are associated with the risk of MSD, to estimate muscular demands. All three parameters have been shown to be useful proxies for WRMSDs [16, 33].

Despite the fact that our analysis evaluated the muscular demand of the trapezius muscle during a routine benign (subtotal) hysterectomy ± cervico-colposacropexy, we assume that muscle activation will be even higher in more complex gynaecological procedures such as laparoscopy for deep infiltrating endometriosis, other prolapse surgeries or pelvic/paraaortic lymphadenectomies. The same can be expected for the flexor carpi radialis muscle.

It has to be stated that the laparoscopic workload per day differs between centres and individuals, however, in higher volume hospitals usually several consecutive laparoscopic procedures are scheduled for one surgeon. As a consequence, we expect a muscular stress that eventually increases over time potentially leading to MSD with emphasis on the shoulder and neck area, especially on the most activated side as demonstrated in our manuscript. Based on the results of our explorative design we see the potential that RALS could be beneficial to decrease symptoms in this area, especially when consecutive operations are performed.

In contrast, it seems obvious that RALS did not reveal benefits for the extensor digitorum muscle as the fingers are constantly activated at the console. At this stage a clear conclusion cannot be drawn from our results for the finger muscles, particularly the left extensor digitorum, however, we hypothesize that in the long term, a potential benefit for the shoulder and neck area in the RALS group may outweigh the increase in muscular demand of the fingers.

### Cardiovascular demands

The median heart rate levels with both techniques were rather low. This is an indicator that the surgeons ensured a routine level of competence for the operations and the applied techniques and that no complications occurred during the analysed procedures. Notably, our findings demonstrate a statistically significant drop in the heart rate during the RALS hysterectomies in contrast to the CLS group. This is possibly due to lower activation of larger muscle groups which have to keep the whole body and arms in a stable standing and working position for laparoscopy. Another debatable reason for a decreased heart rate could be the fact that the console surgeon finds himself in a more enclosed environment compared to the more open setting in CLS where interaction and background noise are contributing factors. It will be interesting to see whether new hybrid robotic systems (the surgeons are mostly positioned at an open console adjacent to the operating table) can also decrease the heart rate significantly. The additional effect of basic physical fitness, surgical challenge and mental stress on the heart rate makes it difficult to further interpret our results as clear cut-off points or scales are missing to draw clear conclusions for the surgeon`s health [34].

### Neck, arm and torso posture

In our analysis, RALS was associated with measurable changes in neck and torso flexion as well as changes in arm abduction and anteversion. This can be well explained by the position of the body and the arms at the console. However, based on the upper quartiles the induced changes in the neck and torso flexion and arm abduction postures during RALS remained within proposed acceptable ergonomic angle positions (acceptable neck flexion area is 0 to 20° neck flexion; acceptable area for torso flexion is 0 to 20° torso flexion, arm abduction 0–20° arm abduction, [35]). Generally, the console can be adjusted to each individual`s head, arm/hand and torso position and can therefore simply meet the proposed acceptable motion ranges. In contrast, the median neck flexion position during CLS was outside the acceptable range which could indicate a poorly adjusted table/monitor position as well as repetitive head movement to focus the introduced instruments/foot pedals during laparoscopy. However, the change in arm anteversion may potentially be negative, since during RALS the arm was in a retroversion position which is considered as non-acceptable. However, it has to be taken into account that the lower arms are supposed to be placed onto the armrest of the console which is currently not addressed by the proposed ranges of acceptable and non-acceptable joint angles [35].

### Perceived workload

Based on previous studies it has been postulated that the mental workload for laparoscopic surgeons is rather high [36]. The decrease in physical demands in one of the six dimensions of perceived workload is in line with the objective muscular demand outcomes described above. However, *mental demands* were statistically significantly increased in RALS. The estimated *task difficulty* of the surgeries as well as the *working precision* were not rated to be statistically different between the two approaches. This finding in mental demands could be a hint that the RALS learning curve of some of the surgeons may not have been fully completed. Depending on the different stages of RALS (e.g. docking, main surgery console time, suturing) various numbers of experiences to achieve a stable learning curve are presented in the literature. Tang reported eight experiences to gain stability for the main surgery console but described 26 experiences to master the suture stage [37]. We defined 10 previous RALS appropriate for inclusion which meets the figures presented by Tang for the main console stage but not for the suture step. It is therefore possible that the low number of previous experiences of some individuals might have an impact both on the perceived workload as well as on the muscular demand in our study. This is one of the limitations of this investigation.

### Musculoskeletal discomfort

Musculoskeletal discomfort was generally rare. Only one surgeon reported discomfort in RALS and CLS. In that case, more discomfort occurred in CLS in the shoulder and lower back, but RALS was associated with finger discomfort which is in line with the literature reporting that the shoulder and neck area may be relieved but the wrist and finger area may be exposed to additional stress compared to CLS [14].

### Potential benefit RALS vs CLS

A review by Hislop and colleagues [32] supports the opinion that RALS is ergonomically superior compared to CLS. However, they point out that this has to be interpreted with caution due to the heterogeneity of studies, multiple sources of bias and small sample sizes. Our present study investigated the ergonomic parameters in five subjects and each of them performed four RALS and four CLS. The sample size is a potential limitation, yet we consider our results a solid basis for further research in this area as the same surgical procedure (benign (subtotal) hysterectomy ± cervico-colposacropexy) was performed in a standardized fashion to reduce heterogeneity and bias.

### Limitations

Published studies with a focus on ergonomic features are mainly based on subjective variables which makes it difficult to draw conclusions that will fundamentally affect the environment for a larger group of surgeons or a subspecialty/discipline. In our study, we, therefore, used three major parameters (muscular demands, heart rate, posture) that could be measured objectively. These objective parameters were completed by the subjective perception of the operation (workload, discomfort). As already discussed, the different levels of the achieved learning curve prior to the study inclusion have the potential to significantly influence muscle, cardiovascular and mental parameters. Due to a relatively low case number with four operations per arm, we also did not allocate the analysed variables to different sex and age groups and did not statistically match patient parameters (e.g. BMI, previous operations, uterus size, difficulty level).

## Conclusion

This study demonstrates a statistically significant ergonomic benefit for RALS versus CLS regarding muscular demands and posture parameters in the analysed areas as well as a significant drop in the heart rate during the procedure. Despite the technically thorough and elaborate design of the investigation, the number of operations per individual were low. We, therefore, consider evaluating our findings of this explorative setting in further studies with hypothesis testing and higher case numbers. Subsequently, this could serve as a database for more complex procedures for which robotics and other assistance systems will have to prove ergonomic advantages and health benefits for the OR staff.

## Supplementary Information

Below is the link to the electronic supplementary material.

Supplementary file1 (DOCX 16 KB)

Supplementary file2 (DOCX 14 KB)

Supplementary file3 (DOCX 13 KB)

## Data Availability

All datasets were generated, analyzed and stored at the Dept. of Women`s Health, Tübingen and at the Institute of Occupational and Social Medicine and Health Services Research, Tübingen.

## References

[1] Epstein S (2018). Prevalence of work-related musculoskeletal disorders among surgeons and interventionalists: a systematic review and meta-analysis. JAMA Surg.

[2] Szeto GP (2012). Surgeons' static posture and movement repetitions in open and laparoscopic surgery. J Surg Res.

[3] Tjiam IM (2014). Ergonomics in endourology and laparoscopy: an overview of musculoskeletal problems in urology. J Endourol.

[4] Alleblas CCJ (2017). Prevalence of musculoskeletal disorders among surgeons performing minimally invasive surgery: a systematic review. Ann Surg.

[5] Ridtitid W (2015). Prevalence and risk factors for musculoskeletal injuries related to endoscopy. Gastrointest Endosc.

[6] Cardenas-Trowers O, Kjellsson K, Hatch K (2018). Ergonomics: making the OR a comfortable place. Int Urogynecol J.

[7] Patel SV (2015). Spin is common in studies assessing robotic colorectal surgery: an assessment of reporting and interpretation of study results. Dis Colon Rectum.

[8] Zizzo M (2022). Robotic versus laparoscopic gastrectomy for gastric cancer: an updated systematic review. Medicina (Kaunas).

[9] Di Donna MC (2022). Conventional laparoscopy versus robotic-assisted aortic lymph-nodal staging for locally advanced cervical cancer: a systematic review and meta-analysis. J Clin Med.

[10] V, Z-M, (2019) Roboterassistierte Chirurgie: Kostenintensiv – bei eher dünner Evidenzlage, in Dtsch

[11] Tarr ME (2015). Comparison of postural ergonomics between laparoscopic and robotic sacrocolpopexy: a pilot study. J Minim Invasive Gynecol.

[12] Mendes V (2020). Experience implication in subjective surgical ergonomics comparison between laparoscopic and robot-assisted surgeries. J Robot Surg.

[13] Armijo PR (2019). Ergonomics of minimally invasive surgery: an analysis of muscle effort and fatigue in the operating room between laparoscopic and robotic surgery. Surg Endosc.

[14] Catanzarite T, Tan-Kim J, Menefee SA (2018). Ergonomics in gynecologic surgery. Curr Opin Obstet Gynecol.

[15] Szeto GP, Straker LM, O'Sullivan PB (2009). During computing tasks symptomatic female office workers demonstrate a trend towards higher cervical postural muscle load than asymptomatic office workers: an experimental study. Aust J Physiother.

[16] Veiersted KB, Westgaard RH, Andersen P (1990). Pattern of muscle activity during stereotyped work and its relation to muscle pain. Int Arch Occup Environ Health.

[17] Alghadir AH (2018). Test-retest reliability, validity, and minimum detectable change of visual analog, numerical rating, and verbal rating scales for measurement of osteoarthritic knee pain. J Pain Res.

[18] Steinhilber B, Caputo G, Seibt R, Rieger MA, Luger T (2020). Influence of a passive trunk exoskeleton on subjective physical strain and perceived discomfort during simulated tasks with static trunk flexion posture and dynamic lifting. Arbeitsmedizin Sozialmedizin Umweltmedizin.

[19] Hart SG, Lowell ES (1988). Development of NASA-TLX (Task Load Index): results of empirical and theoretical research. Adv Psychol.

[20] Kuorinka I (1987). Standardised Nordic questionnaires for the analysis of musculoskeletal symptoms. Appl Ergon.

[21] Kim HY (2013). Statistical notes for clinical researchers: assessing normal distribution (2) using skewness and kurtosis. Restor Dent Endodon.

[22] Field A (2018). Discovering Statistics using IBM SPSS Statistics.

[23] Tamhane N (2020). Robotic surgery for urologic deep infiltrating endometriosis: a review and case presentations. Surg Technol Int.

[24] Smorgick N (2017). Robotic-assisted hysterectomy: patient selection and perspectives. Int J Womens Health.

[25] Go MR (2020). An updated physician workforce model predicts a shortage of vascular surgeons for the next 20 years. Ann Vasc Surg.

[26] Khan S (2020). Inspiring the next generation of surgeons. Postgrad Med J.

[27] van Veelen MA, Jakimowicz JJ, Kazemier G (2004). Improved physical ergonomics of laparoscopic surgery. Minim Invasive Ther Allied Technol.

[28] Gutierrez-Diez MC (2018). A study of the prevalence of musculoskeletal disorders in surgeons performing minimally invasive surgery. Int J Occup Saf Ergon.

[29] Steinhilber B (2017). Ergonomic benefits from a laparoscopic instrument with rotatable handle piece depend on the area of the operating field and working height. Hum Factors.

[30] Steinhilber B (2016). Effect of a laparoscopic instrument with rotatable handle piece on biomechanical stress during laparoscopic procedures. Surg Endosc.

[31] Berguer R (1999). A comparison of forearm and thumb muscle electromyographic responses to the use of laparoscopic instruments with either a finger grasp or a palm grasp. Ergonomics.

[32] Hislop J (2020). Muscle activation during traditional laparoscopic surgery compared with robot-assisted laparoscopic surgery: a meta-analysis. Surg Endosc.

[33] Steinhilber B et al. (2013) Leitlinie Oberflächen-Elektromyographie in der Arbeitsmedizin,Arbeitsphysiologie und Arbeitswissenschaft. Arbeitsmedizinische S2k-Leitlinie der Deutsche Gesellschaft für Arbeitsmedizin und Umweltmedizin (DGAUM) und der Gesellschaft für Arbeitswissenschaft (GfA)

[34] Heemskerk J (2014). Relax, it's just laparoscopy! A prospective randomized trial on heart rate variability of the surgeon in robot-assisted versus conventional laparoscopic cholecystectomy. Dig Surg.

[35] Unfallversicherung, I.f.A.d.D.G., Bewertung physischer Belastungen gemäß DGUV-Information 208–033 (bisher: BGI/GUV-1 7011) (Anhang 3). Deutsche Gesetzliche Unfallversicherung.

[36] Zhang J (2018). Ergonomic assessment of the mental workload confronted by surgeons during laparoscopic surgery. Am Surg.

[37] Tang FH, Tsai EM (2017). Learning curve analysis of different stages of robotic-assisted laparoscopic hysterectomy. Biomed Res Int.

