# Supplementary material for: Save our surgeons (SOS) – an explorative comparison of surgeons’ muscular and cardiovascular demands, posture, perceived workload and discomfort during robotic vs. laparoscopic surgery
Source: Arch Gynecol Obstet. 2022 Nov 19;307(3):849–62. doi: 10.1007/s00404-022-06841-5 (PMC9676911; doi:10.1007/s00404-022-06841-5)
Supplement: Supplementary file 1 — Supplementary file1 (DOCX 16 KB) [file 404_2022_6841_MOESM1_ESM.docx]

| Subject 1 | | Subject 2 | | Subject 3 | | Subject 4 | | Subject 5 | |
| --- | --- | --- | --- | --- | --- | --- | --- | --- | --- |
| Surgical technique | Experimental days | Surgical technique | Experimental days | Surgical technique | Experimental days | Surgical technique | Experimental days | Surgical technique | Experimental days |
| RALS 1 | Day 1 | CLS 1 | Day 1 | RALS 1 | Day 1 | CLS 1 | Day 1 | CLS 1 | Day 1 |
| RALS 2 |  | RALS 1 | Day 2 | RALS 2 |  | RALS 1 | Day 2 | RALS 1 | Day 2 |
| RALS 3 | Day 2 | RALS 2 | Day 3 | RALS 3 | Day 2 | RALS 2 | Day 3 | RALS 2 | Day 3 |
| RALS 4 |  | CLS 2 | Day 4 | CLS 1 |  | CLS 2 |  | RALS 3 |  |
| CLS 1 | Day 3 | RALS 3 | Day 5 | CLS 2 |  | RALS 3 | Day 4 | RALS 4 | Day 4 |
| CLS 2 | Day 4 | CLS 3 | Day 6 | RALS 4 | Day 3 | RALS 4 |  | CLS 2 | Day 5 |
| CLS 3 | Day 5 | RALS 4 | Day 7 | CLS 3 | Day 4 | CLS 3 | Day 5 | CLS 3 |  |
| CLS 4 | Day 6 | CLS 4 | Day 8 | CLS 4 | Day 5 | CLS 4 | Day 6 | CLS 4 | Day 6 |

**Supplemental material A – Order of surgical procedures and number of measurement days**
