# Supplementary material for: Save our surgeons (SOS) – an explorative comparison of surgeons’ muscular and cardiovascular demands, posture, perceived workload and discomfort during robotic vs. laparoscopic surgery
Source: Arch Gynecol Obstet. 2022 Nov 19;307(3):849–62. doi: 10.1007/s00404-022-06841-5 (PMC9676911; doi:10.1007/s00404-022-06841-5)
Supplement: Supplementary file 2 — Supplementary file2 (DOCX 14 KB) [file 404_2022_6841_MOESM2_ESM.docx]

**Supplemental material B - Isometric maximum voluntary contractions for determining the reference activity**

- Left and right descending trapezius muscles: The subject stood upright on a wooden plate, feet hip-width apart, arms in 90° abduction in the scapular plane and elbows were extended. The arm was passed through a metal ring lined with cork and attached to the wooden plate. The exact position of the ring was at the distal part of the upper arm directly before the elbow joint. In this position the ring was chained to the wooden plate and subjects were requested to perform an isometric maximum voluntary (MVC) arm abduction against the resistance of the ring. The MVC was performed twice for the left and right trapezius muscle.
- Extensor digitorum: The subject was seated on a chair with the lower arm resting on a table with the hand palm facing down, hand clenched into a fist, while performing an isometric MVC extension against resistance of one of the experimenters. The MVC was performed twice for the left and right extensor muscle.
- Flexor carpi radialis: The subject was seated on a chair with the lower arm resting on a table with the hand palm facing up, hand clenched into a fist, while performing an isometric MVC flexion against resistance of one of the experimenters.
