# Supplementary material for: Save our surgeons (SOS) – an explorative comparison of surgeons’ muscular and cardiovascular demands, posture, perceived workload and discomfort during robotic vs. laparoscopic surgery
Source: Arch Gynecol Obstet. 2022 Nov 19;307(3):849–62. doi: 10.1007/s00404-022-06841-5 (PMC9676911; doi:10.1007/s00404-022-06841-5)
Supplement: Supplementary file 3 — Supplementary file3 (DOCX 13 KB) [file 404_2022_6841_MOESM3_ESM.docx]

SUPPLEMENT C

**Calculation of neck, arm and torso postures**

| **Joint angle** | **Calculation** | **Interpretation** |
| --- | --- | --- |
| neck flexion | difference between the inclination values (sagittal plane) of the sensor pair placed on the chin and the spinous processes of thoracic vertebrae one (T1) | Positive angles reflect neck flexion, negative angles correspond to neck extension |
| neck lateral flexion | difference between the inclination values (frontal plane) of the sensor pair placed on the chin and the spinous processes of thoracic vertebrae one (T1). | Positive angles reflect neck lateral flexion to the right side, negative angles correspond to neck lateral flexion to the left side |
| arm abduction | difference between inclination values (frontal plane) of the sensor pair placed on the spinous processes of thoracic vertebrae one (T1) and lateral part of the upper arm | Positive angles reflect arm abduction, negative angles correspond to arm adduction |
| arm anteversion | difference between inclination values (sagittal plane) of the sensor pair placed on the spinous processes of thoracic vertebrae one (T1) and lateral part of the upper arm | Positive angles reflect arm anteversion, negative angles correspond to arm retroversion |
| torso flexion | difference between the inclination values (sagittal plane) of the sensor pair placed on the spinous processes of thoracic vertebrae one (T1) and lumbar vertebrae (L4). | Positive angles reflect torso flexion, negative angles correspond to torso extension |
| torso lateral flexion | difference between the inclination values (frontal plane) of the sensor pair placed on the chin and the spinous processes of thoracic vertebra one (T1) and lumbar vertebrae 4 (L4) | Positive angles reflect torso lateral flexion to the right side, negative angles correspond to torso lateral flexion to the left side |
